# Probiotic Encapsulation Technology: From Microencapsulation to Release into the Gut

**DOI:** 10.3390/pharmaceutics4010149

**Published:** 2012-02-06

**Authors:** Gildas K. Gbassi, Thierry Vandamme

**Affiliations:** 1 Laboratoire de Conception et Application de Molécules Bioactives (UMR-7199), Faculté de Pharmacie, UdS-CNRS, 74 Route du Rhin, 67401 Illkirch-Graffenstaden, France; 2 Département de Chimie Générale et Minérale, Faculté de Pharmacie, Université de Cocody, 01 BPV 34, Abidjan, Cote d’Ivoire

**Keywords:** biomaterials, microencapsulation, probiotics, protective device, artificial media, cells release

## Abstract

Probiotic encapsulation technology (PET) has the potential to protect microorgansisms and to deliver them into the gut. Because of the promising preclinical and clinical results, probiotics have been incorporated into a range of products. However, there are still many challenges to overcome with respect to the microencapsulation process and the conditions prevailing in the gut. This paper reviews the methodological approach of probiotics encapsulation including biomaterials selection, choice of appropriate technology, *in vitro* release studies of encapsulated probiotics, and highlights the challenges to be overcome in this area.

## Abbreviations

PET: probiotic encapsulation technology
M: mannuronic acid
G: guluronic acid
FDA: food and drug administration
FAO: food and agricultural organization
WHO: world health organization
CAP: cellulose acetate phthalate
ASM: american society of microbiology
SDS-PAGE: sodium dodecyl sulphate polyacrylamide gel electrophoresis
FTIR-ATR: fourier transformer infra red-attenuated total reflectance
SEM: scanning electron microscope
TEM: transmission electron microscope

## 1. Introduction

Probiotic survival in products is affected by a range of factors including pH, post-acidification during products fermentation, hydrogen peroxide production and storage temperatures [1]. Providing probiotic living cells with a physical barrier against adverse conditions is an approach currently receiving considerable interest [2].

Probiotic encapsulation technology (PET) is an exciting field of biopharmacy that has emerged and developed rapidly in the past decade. Based on this technology, a wide range of microorganisms have been immobilized within semipermeable and biocompatible materials that modulate the delivery of cells. The terms immobilization, entrapment and encapsulation have been used interchangeably in most reported literature [3]. While encapsulation is the process of forming a continuous coating around an inner matrix that is wholly contained within the capsule wall as a core of encapsulated material, immobilisation refers to the trapping of material within or throughout a matrix [3]. Encapsulation tends to stabilize cells, potentially enhancing their viability and stability during production, storage and handling. An immobilized environment also confers additional protection to probiotic cells during rehydration. As the technique of immobilization or entrapment became refined, the cell immobilization technology has evolved into cell encapsulation technology [3], which we refer to here as PET.

The best application of PET in biopharmacy is the controlled and continuous delivery of cells in the gut. The potential benefit of this therapeutic strategy is to maintain greater cell viability despite the acidity into the stomach. In their viable state, probiotics may exert a health benefice on the host [4,5]. One research group showed that alginate could pass through the stomach without any degradation. Gel beads formed from this biomaterial were visualized in the human gut by nuclear magnetic resonance imaging [6]. The choice of the biomaterial is crucial because it determines the effectiveness of the protective device. Beyond this protection, the device must withstand during the passage through the stomach, disintegrate in the gut to release the cells. Probiotics are currently encapsulated in polymer matrices for various applications. The physical retention of cells in the matrix and their subsequent separation is the consequence of the encapsulation technology used.

Selecting the encapsulation technology is very important. Whereas probiotics are living cells, the conditions for implementation of this technology are designed to maintain cell viability, and solvents involved in the encapsulation technology must be non-toxic. Furthermore, assess the release conditions of encapsulated probiotics in a gastrointestinal tract model is an essential approach, which would give an idea of the cells’ behavior.

This paper reviews the methodological approach of probiotics encapsulation including biomaterials selection, choice of appropriate technology, *in vitro* release studies of encapsulated probiotics, and highlights the challenges to be overcome in this area.

## 2. Selecting the Biomaterials for Microencapsulation

The concept of biomaterials usually results in various definitions. A definition often accepted in the field of biology and medicine is “any natural material or not, which is in direct contact with a living structure and is intended to act with biological systems” [7]. The biomaterials used for probiotics encapsulation include natural polymers and synthetic polymers [7]. The terms biocompatible and biodegradable are associated with many of these biomaterials. Biomaterials for probiotics encapsulation are in direct contact with the living cells.

After microencapsulation, the protective device-based biomaterial is intended to be in contact with the digestive tract of the host. For all these reasons, much of the general criteria developed for choosing biomaterials can be applied. Issues involved when selecting biomaterials for probiotics encapsulation are: (a) physicochemical properties (chemical composition, morphology, mechanical strength, stability in gastric and intestinal fluids; (b) toxicology assay; (c) manufacturing and sterilization processes.

Biomaterials are inorganic or organic macromolecules, consisting of repeated chain of monomers linked by covalent bonds. Their chemical structure and the conformation of the monomer chains give them specific functionality such as ability to form gels [8]. The most common biomaterial used for probiotics encapsulation is alginate [9,10,11]. Other supporting biomaterials include carrageenan, gelatin, chitosan, whey proteins, cellulose acetate phthalate, locust bean gum and starches [11].

Alginate is a linear polymer of heterogeneous structure composed of two monosaccharide units: acid α-L-guluronic (G) and acid β-D-mannuronic (M) linked by β (1–4) glycosidic bonds [12,13]. The appearance of G and M monomers in the alginate chains occurs in blocks of alternating sequences, not randomly. This arrangement of chains is widely described in the literature and varies from one structure to another [13,14,15,16]. The M/G ratio determines the technological functionality of alginate. The gel strength is particularly important that the proportion of block G is high. Temperatures in the range of 60 °C to 80 °C are needed to dissolve alginate in water. Alginate gels are known to be insoluble in acidic media [17]. The success of the use of alginate in microencapsulation of probiotics is due to the basic protection against acidity it provides to the cells [18,19,20].

Carrageenan are polymers of linear structure consisting of D-galactose units alternatively linked by α(1–3) and β(1–4) bonds. Three types of carrageenan are known: kappa (κ) carrageenan, iota (ι) carrageenan and lambda (λ) carrageenan [21]. κ-Carrageenan (monosulfated) and ι-carrageenan (bisulfated) have an oxygen bridge between carbons 3 and 6 of the D-galactose. This bridge is responsible for conformational transitions. It is also responsible for the gelation of κ-carrageenan and ι-carrageenan. The λ-carrageenan (trisulfated) that does not have this bridge is unable to gel [22]. Carrageenan gelation is induced by temperature changes. A rise in temperature (60 to 80 °C) is required to dissolve it and gelation occurs by cooling to room temperature [22,23]. Carrageenan is commonly used as food additive; its safety has been approved by several government agencies including FDA, codex alimentarius and the joint FAO/WHO food additives [24]. The use of carrageenan in microencapsulation of probiotics is due to its capacity to form gel that can entrap the cells. However, the cell slurry should be added to the heat-sterilized suspension between 40 and 45 °C, otherwise the gel hardens at room temperature [25].

Whey proteins are usually used because of their amphoteric character. They can be easily mixed with negatively charged polysaccharides such as alginate, carrageenan or pectin [25,26]. When the pH is adjusted below their isoelectric point, the net charge of the proteins becomes positive, causing an interaction with the negatively charged polysaccharides [17,27,28].

Gelatin is frequently used in the food and pharmaceutical industries. It is a protein derived by partial hydrolysis of collagen of animal origin. Gelatin has a very special structure and versatile functional properties, and forms a solution of high viscosity in water, which sets to a gel during cooling [29]. It does not form beads but could still be considered as material for microencapsulation.

Chitosan is a positively charged polysaccharide formed by deacetylation of chitin. Its solubility is pH-dependent. It is water insoluble at a pH higher than 5.4 [30]. This insolubility presents the drawback of preventing the complete release of this biomaterial into the gut which pH is greater than 5.4 [30]. However, studies have reported the effectiveness of chitosan as a coating agent of alginate gel beads [30,31,32]. Chitosan can form a semipermeable membrane around a negatively charged polymer [29]. Whey proteins, gelatin and chitosan are usually used to develop capsules [9] or to coat gel beads to improve their stability [11].

Cellulose acetate phthalate (CAP) is a polymer insoluble at a pH below 5 but and soluble when the pH is greater than 6 [9,11]. This property is essential for probiotics encapsulation because the biomaterial must not dissolve into the stomach, but only into the gut. The disadvantage of CAP is that it cannot form gel beads by ionotropic gelation; only capsules have been developed by emulsification using this biomaterial. CAP is widely used as a coating agent.

Locust bean gum and starches are usually mixed with alginate or carrageenan to develop gel beads or capsules. It appears that specific interactions occur during mixing. The ratio between the proportions of each biomaterial before mixing is essential [9].

Selecting the appropriate biomaterial is a preliminary study which requires a rigorous methodological approach. For probiotics encapsulation, biomaterials such as proteins and polysaccharides must be stable in acidic environment and unstable in environment with a pH above 6. This pH is the minimum pH found in the intestinal lumen, usually at the beginning of the duodenum [18]. For example, the stability of proteins under varying conditions of pH can be assessed by electrophoresis (SDS-PAGE). For polysaccharides and other biomaterials treated under various conditions of pH, FTIR-ATR can be used to study their stability by determining the any change in its initial structure. Publications have referred to the mixture of biomaterials (proteins-polysaccharides or polysaccharide-polysaccharide) to encapsulate probiotics [1,2]. However, it would be interesting to elucidate the interactions between these biomaterials [17]. Once the biomaterial has been used to develop the protective device, it would also be interesting to elucidate the mechanism of resistance of this device in an acidic medium, and its disintegration or dissolution in environment with a pH above 6. Searching for new encapsulation materials will be of paramount importance in the near future. These materials must meet the requirements of non-toxicity, resistance to gastric acidity and compatibility with respect to probiotic cells. Several challenges are faced in this area.

## 3. Selecting the Microencapsulation Technology

Most of the reported literature on PET was based on small-scale laboratory procedures. PET requires techniques that are gentle and non-aggressive towards the cells. The first encapsulation techniques developed to improve the shelf-life of probiotics were to transform cells cultures into concentrated dry powder. The techniques of spray-drying, freeze-drying or fluidized bed drying have shown their limitations because the cells encapsulated by these techniques are completely released into the product. Thereby, the cells are not protected towards the food matrix environment and in the presence of gastric fluid or bile [33]. However, probiotics in dried or freeze-dried form exhibit compatibility with traditional starter culture such as milk or cheese and have a longer shelf-life compared to their cell slurry form [29].

With specific reference to spray-drying, recent publications make reference to its effectiveness in protecting probiotic cells [34,35]. This technique commonly used in food industry involves atomization of an aqueous or oily suspension of probiotics and carrier material into a drying gas, resulting in rapid evaporation of water [29]. Water evaporation is defined as the difference between air inlet temperature and air outlet temperature. The spray-drying process is controlled by these temperatures, but also by the product feed and the gas flow [29]. Despite the advantages of spray-drying technique, the high temperatures needed to facilitate water evaporation reduce the probiotics viability and their activity in the final product. The minimum air inlet temperature reported in the literature for probiotic encapsulation is 100 °C while the maximum is 170 °C. The air outlet temperature vary between 45 °C and 105 °C [29]. At these temperatures, it is unlikely that the cells retain all their probiotic activity. Probiotic activity must be differentiated from probiotic survival. Probiotic activity takes into account the ability of cells to resist to gastrointestinal environment and to adhere to intestinal mucosa [36], so it is important that the encapsulation technique does not reduce cell survival and does not inhibit their subsequent activities.

Providing probiotics with a physical barrier against adverse conditions is an approach receiving considerable interest. For this, other techniques have been introduced to further improve the protection of probiotics. These techniques were intended to develop gel beads or capsules which were made from hydrocolloids by means of extrusion or emulsification techniques [37,38]. Hydrocolloids are aqueous dispersion of biomaterials (natural or synthetic polymers).

**Figure 1:**
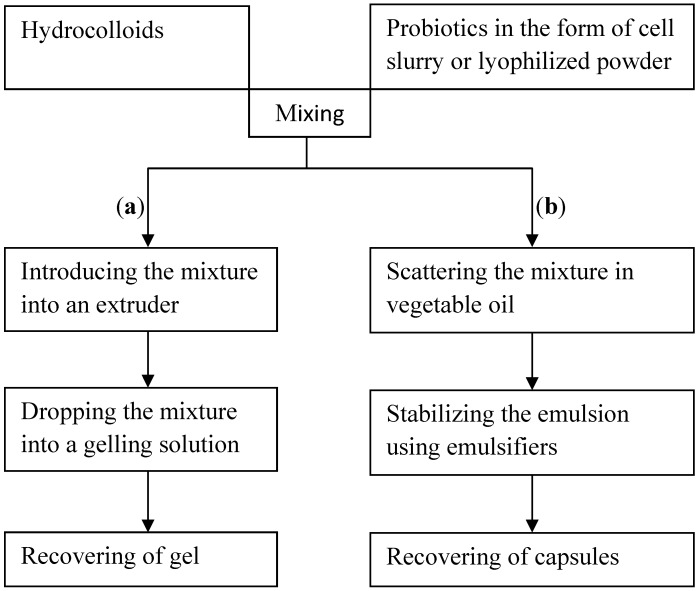
Diagram of the encapsulation process of probiotics by extrusion technique (**a**) and by emulsification technique (b).

The encapsulation process of these two techniques is summarized in Figure 1.

In extrusion technique (a), the hydrocolloid is mixed with probiotics. The resulting mixture is fed into an extruder, typically a syringe. Pressure exerted on the syringe plunger drops the contents of the syringe into a gelling solution, with gentle stirring. The size and shape of the drops depend on the diameter of the needle, and the distance between the needle and the gelling solution. Extrusion is a simple and easy implementation, allowing the retention of a high number of cells. Automated processes exploiting this principle are available today [39].

In the emulsification technique (b), the mixture represents the discontinuous phase. This phase is dispersed in a large volume of vegetable oil (continuous phase). The water-in-oil emulsion being formed is continuously homogenized by stirring. The stirring speed is a critical step because it affects the size and the shape of the droplets formed [40]. Once the emulsion has been broken, the droplets are collected by settling. The use of this technique for probiotics encapsulation has been described in the literature [40,41].

**Figure 2:**
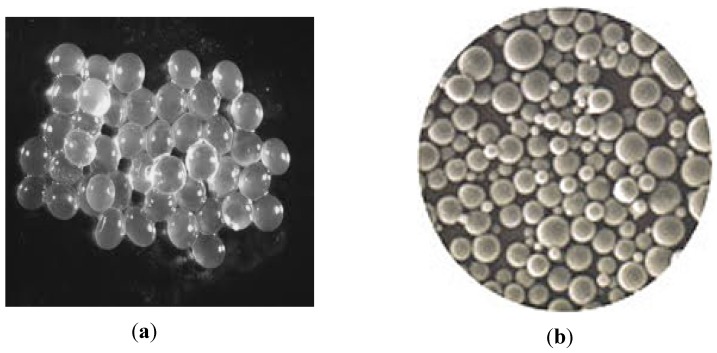
(**a**) Photographs of alginate gel beads and (**b**) Photographs of alginate capsules [39].

Emulsification generates oily or aqueous droplets commonly named capsules, while the extrusion gives gelled droplets called beads. The core of the capsule is liquid while the core of the bead presents a porous network [7]. The capsules have sizes that are at least 100 times lower than those of the beads [9]. The difference between capsules and beads is shown in Figure 2. Capsules have unequal size and shape compared to beads whose shape is uniform.

Extrusion is much easier to realize compared to emulsification. Emulsification is more expensive because it requires additional raw materials such as vegetable oil and emulsifiers to stabilize the emulsion. Emulsification also presents difficulties in implementation including emulsion instability, need for vigorous stirring which can be detrimental to cells survival, random incorporation of cells into the capsules, and inability to sterilize vegetable oil if you have to work under conditions of strict asepsis.

From these two techniques are introduced changes to improve beads or capsules stability. Among these improvements are coating with others biomaterials [32], cross-linking with organic solvents [42], or adding additives or cryoprotectants in the mixture [43]. In the literature, rare are the studies in which authors have shown photographs of probiotics entrapped in capsules. Electron microscopy (SEM or TEM) is an effective technique to provide evidence of the presence of probiotics in capsules or beads and to assess the bacterial loading [20].

## 4. Selecting the *in Vitro* Conditions for Cells Release

When probiotics are encapsulated, it is essential to check two conditions. First, ensure that the protective device of probiotics is reliable in media simulating the gastric fluid, and then ensure that the encapsulated probiotics are released in media simulating the intestinal fluid.

In the literature, experimental models simulating the gastro-intestinal tract have been described. These models evaluate the tolerance of probiotics to acidic media, bile and enzymes. There are generally two types of experimental models, known under the names of “conventional model” and “dynamic model”. The dynamic model differs from the conventional model because it is semi-automated. Different approaches have been proposed. The conventional model simulates either the stomach or the gut. It consists of a single reactor (glass container) containing the simulated gastric fluid or the simulated intestinal fluid. The dynamic model consists of a series of reactors with respective volume for stomach and gut, in which the temperature was maintained at 37 °C and the pH was automatically controlled to maintain values of gastric and intestinal pH. All reactors were continuously stirred, and the sterile culture medium was fed to gastric reactor by a peristaltic pump which sequentially supplied the gut reactor. Flow rate was set to obtain the mean transit time throughout the model [44,45,46,47].

The *in vitro* conditions used for the simulation of the stomach are detailed in Table 1.

**Table 1:** *In vitro* conditions most often used to simulate the stomach.

Gastric fluid	pH values	Pepsin content (g/L)	Exposure time (min)	References
NaCl (2 g/L)	1.55	0	180	[18]
	2 and 3	0	120	[19]
	1.55	0	120	[32]
	2	0	60	[48]
NaCl (5 g/L)	2	3	60	[49]
	2	3	180	[50]
	2 and 3	3	240	[51]
NaCl (8.5 g/L)	2.5	3	90	[52]
	2 and 3	3	90	[53]
	2	0	120	[40]
NaCl (9 g/L)	1.8	3	120	[20]
HCl (3.65 g/L)	1.1	0	120	[54]
	1.9	0.26	30	[55]
	2 and 3	0	120	[56]
MRS broth (55 g/L)	2	0	120	[57]
Peptone broth (7.5 g/L)	2 and 3	0.3	20	[58]
Cheese broth (8.5 g/L)	2.5 and 3	0.016	120	[59]
	2 and 3	0	180	[60]
Skimmed milk (12 g/L) glucose (2 g/L) yeast extracts (1 g/L) and cysteine (0.05 g/L)	2 and 3	0	60	[10]
	2 and 3	0	180	[41]
Glucose (3.50 g/L) NaCl (2.05 g/L) KCl (0.37 g/L) KH_2_PO_4_ (0.60 g/L) CaCl2 (0.11 g/L) porcine bile (0.05 g/L) and lysosyme (0.10 g/L)	2	0.013	90	[61]

When reading the Table 1, a preference for the NaCl medium was noted. More than half of the authors have suggested this. However, concentrations of 2 and 5 g/L of NaCl used seem insufficient to maintain the isotonicity of the medium. The American society of microbiology (ASM) recommends saline solution at 9 g/L in the microbiological procedures such as microbial cells suspension or dilution, and tolerance tests to antimicrobial substances [62]. NaCl provides an isotonic medium that maintains the integrity and the viability of the microbial cells. The ASM also reported that phosphate can be added to NaCl medium to buffer it. In this case, the concentration of NaCl should be reduced (8 to 8.5 g/L). Phosphate addition provides a stable pH because of its buffering capacity, which helps to maintain cell viability.

Regarding the gastric fluid pH, it should be noted that the values vary between 1 and 3. This pH range covers the values generally observed in human’s stomach [63]. Pepsin was sometimes used as a model of gastric enzyme. However, no information is yet available about the true concentration of this enzyme in the stomach. This reflects the fact that pepsin is secreted in the form of pepsinogen (inactive form) which is then activated in pepsin by the presence of acidic medium [64]. Pepsin activity requires a pH under 5.6 [64,65]. Any artificial gastric fluid must include this enzyme in its composition.

Finally, regarding the exposure time, several values were observed, ranging from 20 min to 240 min. However, clinical studies have shown that a period of 120 min was sufficient to ensure the gastric emptying of 90% of a liquid meal [66] and 60% of a semi-solid meal [66,67,68]. An exposure time of 120 min is reasonable for the stay of probiotics in an artificial gastric medium. After a stay of probiotics in the stomach, the gut is naturally the second favorite place, so tests are conducted in this part of the gastro-intestinal tract.

The Table 2 presents the *in vitro* conditions used for the simulation of the gut.

**Table 2:** *In vitro* conditions most often used to simulate the gut.

Intestinal fluid	pH values	Bile (g/L)	Enzymes (g/L)Pancreatin Trypsin		Exposure time (min)	References
NaHCO_3_ (25.2 g/L)	6.5	40	3.5	0.1	240	[47]
NaCl (5 g/L)	8	45	1	0	180	[50]
Na_2_HPO_4_ (2.84 g/L)	7.5	150	1.95	0	360	[55]
PBS* (1 mol/L)	8	1	1	0	180	[58]
PBS (np**)	7.4	2	1	0	180	[69]

* Phosphate Buffer Saline ** Unspecified. PBS defines a medium composed of various salts whose proportions vary from one author to another.

Bile and pancreatic enzymes are present in the lumen of the gut [70,71], so only studies involving the presence of bile and at least one pancreatic enzyme have been emphasized in this review. When reading the Table 2, sodium salts are exclusively used as intestinal fluid at various concentrations. The term PBS refers to a phosphate buffered saline. In reality, it consists mainly of NaCl in which other salts were added: NaCl (8.5 g/L), K_2_HPO_4_ (1.1 g/L) and KH_2_PO_4_ (0.32 g/L) [72]. Sometimes it consists of NaCl (8 g/L), Na_2_HPO_4_ (1.44 g/L) and KH_2_PO_4_ (0.24 g/L) [62]. One author used it incorrectly to refer to an aqueous solution containing only sodium chloride [40]. In many cases, the composition of PBS was not mentioned [58,69]. Moreover, it can be a medium in which the salt concentrations have been adjusted or supplemented by other salts as needed [73].

The pH values used are between 6.5 and 8. These values reflect the pH usually met in the gut [74]. Regarding the concentrations of bile and enzymes, no published data allows specifying the exact levels, which may explain the variations observed from one author to another. The lack of published data on the transit time of the gut may explain the difference observed in the exposure time. Studies with radio-labeled food must be conducted to determine this transit time.

The studies summarized in Table 1 and Table 2 clearly show a lack of standard protocol in establishing the *in vitro* conditions for simulating the stomach or the gut. Searching a consensus in the standardization of protocols must be in compliance with the conditions prevailing into the gastro-intestinal tract. Type of medium and its composition, choice of pH values, exposure time, presence of gastric or intestinal enzymes, and presence of bile are the essential factors to be taken into account. These factors should reflect reality as much as possible in humans.

## 5. Conclusion and Future Perspectives

PET is widely described in the literature. Since its emergence in the 1990s, tremendous advances have been made in this field. PET has been constantly improved, modified and adapted. Despite these developments, there are still many challenges in this area, such as developing microencapsulation equipment, clarifying microencapsulation procedures, choosing non-toxic materials for probiotics encapsulation, developing capsules or beads from polymers adapted to the pH of the digestive tract, determining mechanisms of probiotics release from capsules or beads, carrying out *in vitro* and *in vivo* studies and assessing microencapsulation costs. Many challenges are yet to be overcome and PET seems to be not yet well developed, as has been discussed by [2] and [29].

The challenge of equipment refers to beads or capsules sizes, which are crucial and should be carefully controlled. Small capsules or beads under controlled conditions will not affect the texture of food products [29]. Most of the procedures of PET reported involve emulsification technology and extrusion technology (also called ionotropic gelation). In emulsification technology, emulsifier or surfactant added in vegetable oil was used to promote the capsule. This technique may not be suitable for food product development because the residual oil in the encapsulated material is detrimental to texture and organoleptic characteristics, and may not be suitable for the development of low-fat dairy products [1]. The residual oil, emulsifier and surfactant in the encapsulated material can be toxic to probiotic cells and may interact with food components [29]. The resulting capsules are considered to be not uniform (Figure 2). This can affect mouth feel and will therefore not be suitable for incorporation into food [1]. Research needs should lead to the development of microcapsules using only aqueous gelling without use of emulsifier, surfactant or oil. In terms of handling conditions and safety requirements, extrusion seems better to probiotics encapsulation. However, extrusion will face the challenge of large-scale production of beads [32]. PET has been applied to dairy products such as yogurt, milk, frozen dessert and cheese. The selection is now expanding to fruit juices, cookies and chocolate [29]. Recognition of new applications in which food matrices may interact with encapsulated probiotics requires additional experimental work. Companies using PET need further expertise to be able to estimate the most promising commercial applications.

Another challenge will be to determine the physicochemical characteristics of encapsulation materials to predict their mechanisms of disintegration or dissolution under varying conditions of pH and salinity and their interactions with probiotic cells or other components present in the digestive tract. PET will be of importance in delivering viable strains of probiotic to consumers in the near future. Evidence of this delivering must firstly be provided by the results of *in vitro* studies, through simulation of simple and reproducible gastrointestinal tract models. At this level, the lack of standard protocol in the conduct of these tests remains a concern. Efforts should be made in this direction by the scientific community. A model of gastro-intestinal tract has been recently published by Gbassi *et al*. [75]. This model regarding its principle and its implementation can serve as a framework for reflection in order to understand all aspects of protocols standardization.

Clinical data resulting from *in vivo* studies will confirm the delivering of probiotics in the gut, but also provide evidence of their health benefits. Legislation in the United State of America allows probiotics under dietary supplement health [1]. In Europe, probiotics are defined by their application: drug or food [76]. Probiotics used as dietary supplements or functional foods are regulated by food legislation. A positive list of health claims with their conditions of use is defined. For any drug claim, scientific evidence of the health benefits must be provided.

The final challenge is to minimize the costs of PET. According to [29], the development of value-added products such as encapsulated end products will have higher prices. Since product development takes both time and financial resources, the microencapsulation phase of probiotics adds additional costs to food processing. The costs may vary greatly depending on the technique used and the volume of the product. Encapsulation using natural polymers (polysaccharides and proteins) are expensive [11,39] and milk proteins are more costly than carbohydrates. The emulsification technique is more expensive because it requires additional raw materials such as oil and emulsifiers to stabilize the capsules [32]. Spray chilling, rarely reported for probiotics, is considered the least expensive encapsulation technology [39]. PET has great potential for the future if the challenges identified are resolved by scientists and industrialists.
